# Magnetic Field Sensing Based on Bi-Tapered Optical Fibers Using Spectral Phase Analysis

**DOI:** 10.3390/s17102393

**Published:** 2017-10-20

**Authors:** Luis A. Herrera-Piad, Joseph W. Haus, Daniel Jauregui-Vazquez, Juan M. Sierra-Hernandez, Julian M. Estudillo-Ayala, Yanelis Lopez-Dieguez, Roberto Rojas-Laguna

**Affiliations:** 1 Departamento de Ingeniería Electrónica, División de Ingenierías, Universidad de Guanajuato, Carretera, Salamanca-Valle de Santiago km 3.5 + 1.8, Comunidad de Palo Blanco, Salamanca Gto. C.P. 36885, Mexico; la.herrerapiad@ugto.mx (L.A.H.-P.); jm.sierrahernandez@ugto.mx (J.M.S.-H.); julian@ugto.mx (J.M.E.-A.); y.lopezdieguez@ugto.mx (Y.L.-D.); rlaguna@ugto.mx (R.R.-L.); 2 Department of Electro-Optics and Photonics, University of Dayton, Dayton, OH 45469, USA; jhaus1@udayton.edu

**Keywords:** tapered optical fibers, magnetic fiber optic sensor, optical signal processing

## Abstract

A compact, magnetic field sensor system based on a short, bi-tapered optical fiber (BTOF) span lying on a magnetic tape was designed, fabricated, and characterized. We monitored the transmission spectrum from a broadband light source, which displayed a strong interference signal. After data collection, we applied a phase analysis of the interference optical spectrum. We here report the results on two fabricated, BTOFs with different interference spectrum characteristics; we analyzed the signal based on the interference between a high-order modal component and the core fiber mode. The sensor exhibited a linear response for magnetic field increments, and we achieved a phase sensitivity of around 0.28 rad/mT. The sensing setup presented remote sensing operation and low-cost transducer magnetic material.

## 1. Introduction

For several decades, the optical fiber sensor community has spent its efforts proposing many structures and methods to measure physical parameters. Two main measurement modalities are available: phase and intensity. Both methods have pros and cons. For instance, intensity modulation has a simple demodulation process, but the measurement can be affected by source variations and environmental effects around the fiber. On the other hand, phase modulation exhibits immunity to intensity variations, but unfortunately its demodulation signal requires additional analysis, and there may be phase ambiguity due to phase wrapping. In addition, there are other points to consider in fiber optic sensors such as the reproducibility of the fabrication process and insertion losses affecting the signal-to-noise ratio. 

Some of the first attempts to detect the magnetic field consisted in analyzing the polarization effects and the intensity changes generated by deforming a single mode fiber using an external sensitive magnetic material [1,2,3]. These works use an external material that responds when a magnetic field is applied. Indeed, contemporary works combine special materials with Fabry–Perot interferometers (FPIs) [4,5] or linear fiber lasers [6] to propose a magnetic field sensor. Nevertheless, in recent years, nanoparticles with special magnetic properties have been studied. These nanoparticles, merged with a liquid, provide a magnetic fluid that, combined with fiber optic structures, generate a magnetic fiber optic sensor; these structures are tapered optical fibers [7,8,9,10,11,12,13], FPIs [5,14,15,16,17,18], ring fiber lasers [19,20,21], fiber Bragg gratings [22,23,24,25,26], multi-mode interferometers [27], core-offset interferometers [28], long period gratings [29,30], and filled fiber structures [31,32]. As can be appreciated, the magnetic optical fiber sensors are strongly related to the material involved, many of them are expensive or not commercially available. In addition, most of the above mentioned works measure the phase modulation to estimate the magnetic field, but other groups measure the intensity modulation [25,26]. In the latter case, the optical fiber undergoes a chemical treatment that increases the losses in the system when a magnetic field is applied. Nevertheless, intensity modulation has certain advantages: moreover, the fabrication procedure proposed is more complex, and a special temperature control system is required.

In this work, we propose an alternative signal processing method to detect any physical parameter that presents phase modulation. To validate the processing signal method, we present a study of a magnetic field fiber optic sensor based on a bi-tapered optical fiber (BTOF). Here, the design of a BTOF, operated in reflection mode, laid over an inexpensive thin magnetic tape as a magnetic field fiber optic sensor. Our design is compact, requiring only a centimeter fiber span, and is insensitive to signal polarization changes, making it suitable for applications outside the laboratory environment. We apply a magnetic field across a range of 30 mT using a permanent magnet that can be moved close to the magnetic tape. The tape bending, induced by the magnetic field, generates phase modulation of the optical signal. From our experiments with specific bi-tapered fiber design parameters, we can extract a maximal wavelength sensitivity around 70 pm/mT and the signal analysis shows low response linearity. We show that this issue can be overcome via phase analysis. As previously demonstrated [33,34], the spatial frequency analysis represents an alternative method of exploiting the fiber optic sensor’s high sensitivity. In our case, we use this technique to improve the detection and the linearity response. Here, the phase analysis offers a better linear response (R^2^ = 0.98) and sensitivity (~0.28 rad/mT). 

## 2. Experimental Setup and Principle Operation

**Figure 1:**
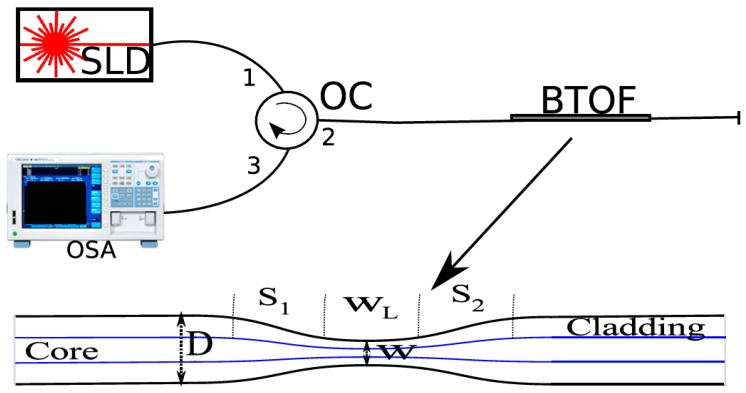
Sketch of the experimental setup (www.yokogawa.com): SLD: super luminescent diode; OSA: optical spectrum analyzer; OC: optical circulator; BTOF: bi-tapered optical fiber. The bottom inset illustrates the BTOF geometry (up- and down-taper sections: S_1_ and S_2_; waist length: W_L_; waist diameter: W).

Our experimental setup consists of three main elements: a super luminescent diode (SLD: QSMD-1550-1,), an optical spectrum analyzer (OSA: Yokogawa AQ6370B, Co., LDT, Newman, GA, USA), and a BTOF.We designed our system using standard single-mode optical fibers; the BTOF operates in reflection mode, the aforementioned elements were interconnected using an optical fiber circulator (see Figure 1). The BTOF operation is based on the interference between fiber modes in the waist region. A single mode fiber is down-tapered (S_1_) to launch power into two or more modes in the waist region; each mode acquires a different phase as it crosses the waist; when the mode recombine to a single mode in the up-taper section (S_2_) they interfere constructively or destructively and overlap with the single core mode. As the wavelength is changed, the phase difference between two modes periodically changes [34]. 

**Figure 2:**
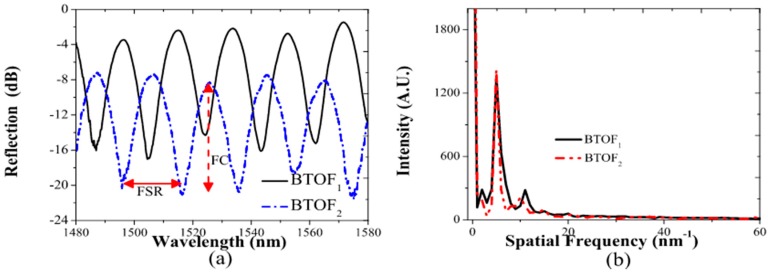
(**a**) Reflection spectrum of the fabricated BTOFs (BTOF1 and BTOF2); (**b**) spatial frequency spectrums of the BTOFs.

Moreover, to validate our proposed method, two BTOFs with quasi-adiabatic tapers were fabricated by the flame brushing technique. In this technique, a length of single mode fiber (8 μm core/125 μm diameters) without plastic protection was held at each end by translation stages that were motorized. A butane flame is scanned back and forth along the fiber length; at the same time the two motorized translation stages simultaneously stretch the fiber [35,36]. It is important to stress that important parameters such as flame position and intensity as well as the motors’ pull tension and flame scan rate velocity are computer-controlled. For an exponential decrement of the diameter in zones S_1_ and S_2_, the radii at position z can be expressed by $r\left(z\right)=r_{c}e^{-z/2L_{0}}$, with the taper length L satisfying r(L)=7 μm and $r_{c}=62.5\;\mu m$, and $L_{0}=2\;\mathrm{mm}$ is the length of the initial flame heated section of the fiber [37]. These structures have the same physical dimensions (see Figure 1 inset): a quasi-adiabatic region of 5 mm (down- and up-taper sections S_1_ and S_2_), a waist around 14 μm (W), and a waist length of 2 mm (W_L_). As is well known, reproducibility is a drawback of the fabrication process used in this work. In our particular case, two BTOFs (BTOF_1_ and BTOF_2_) were intentionally fabricated using the same process, but different insertion losses (BTOF_1_ = 2 dB and BTOF_2_ = 7 dB), and a similar free spectral range (FSR) (19.59 nm) with a high fringe contrast (FC) around 11.54 dB, were obtained. The variations of the insertion losses are related to the strain differences applied to the fibers during the fabrication process when a length of fiber is set between two translation motors. Nevertheless, the fabrication error is small and the FSR is the same, and only the losses between two different fibers are found. In other words, the amplitude changes do not affect the dominant Fourier component of the interference spectrum [38]. The wavelength spectrums of both samples exhibit different absolute signal phases, but the analysis does not require them to be the same since each fiber is self-referenced for the signal phase analysis (see Figure 2). 

Fourier transform signal analysis was applied to the wavelength reflection spectrums of the BTOFs; their spatial frequency amplitudes are shown in Figure 2b. Despite the power level disparity and the difference phase-matching observed in the reflection interference (around π), the spatial frequency spectrum shows similar modal contribution. Both (spatial) frequency spectrums exhibit the presence of two, dominant signal frequencies: the component centered at zero frequency (D.C. component) and the interference between a high-order (cladding) mode and the core mode with a peak centered around 5 nm^−1^. As can be observed in Figure 2b, the dominant spatial frequencies for both BTOFs are close to one another and their energy content, based on the peak heights and widths, are also very similar. These frequency components are used to implement a magnetic field phase-sensitive analysis. 

The interference reflection spectrums of the BTOFs (see dot-dash and solid lines in Figure 2a) generate the strong peaks observed in Figure 2b. The interaction between them can be expressed by the following expression:

(1) $$I=I_{a}+\overset{}{\underset{b}{\sum }}I_{b}+2\overset{}{\underset{b}{\sum }}\sqrt{I_{a}I_{b}}cos\;\left(\Delta \phi_{ab}\right)\;$$

where *I_a_* represents the core mode intensity, and *I_b_* is the intensity of the cladding modes excited in the waist region of the BTOFs. The first two terms in Equation (1) represent zero frequency contributions. The differential phase components between the modes involved is represented by $\Delta \phi_{ab}$. This difference phase is related to the difference in propagation constants: $\beta_{b}-\beta_{a}$ where $\beta_{a,b}=2\pi n_{a,b}L/\lambda$, where λ is the wavelength, *L* is the total length of the BTOFs, and *n_a,b_* represents the effective refractive index of the core mode (*n_a_*) and high-order cladding modes (*n_b_*) in the waist. The effective refractive index is related to the BTOF dimensions and is strongly wavelength dispersive.

## 3. Magnetic Field Detection

### 3.1. Waveleght Analysis

**Figure 3:**
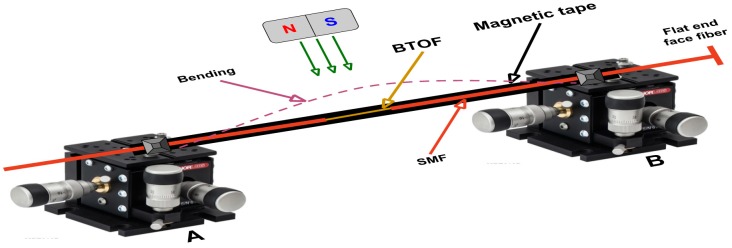
Schematic diagram of the experimental setup. The magnetic field is changed by moving a permanent magnet close to or further from the magnetic tape (www.thorlabs.com).

As a proof of principle for the magnetic field sensor, our BTOF structures were laid over a commercial thin plastic magnetic tape (length: 11.5 cm, width: 12.7 mm and thickness: 25.4 microns), and the principal magnetic element in the magnetic tape is a well known Fe_3_O_4_ material. The ends of the magnetic tape and the BTOF were fixed using two translations stages (A and B). The magnetic field was applied using a magnet attached to a vertical translation stage. When the distance between the magnet and magnetic tape decreased, the magnetic field over the magnetic tape surface increased, and the magnetic tape with the attached BTOF bowed toward the magnet (see Figure 3). The magnetic field was estimated using a Magnetometer E-1008537, and the maximum magnetic field applied was 59 mT and the maximal curvature produced was 2.4 m^−1^. As the magnet was lowered toward the tape, the tape’s bowing curvature above the platform increased. The motion of the tape changed the fiber curvature, and the reflection spectrum was shifted to longer wavelengths. The wavelength shift is related to the effective refractive index changes generated by the bending induced over the BTOF [39]. To prevent temperature effects from affecting our magnetic field detection system, the experiment was kept at room temperature, 25 °C. Other authors have reported inducing a magnetic field using current flow through a metal wire coil, which requires a special system to measure the temperature effects at the sensor or to keep its temperature constant [25,26]. We eliminated the undesirable local heating effects using a permanent magnet in our experiments and directly measured the local magnetic field.

**Figure 4:**
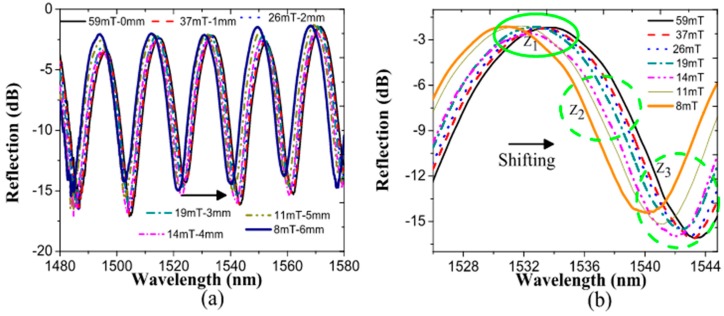
(**a**) Magnetic field response for specific magnet positions; (**b**) Particular fringe regions analyzed for magnetic field increments. Z_1_ is near the signal maximum, Z_2_ is where the signal’s slope change is the highest, and Z_3_ is at the signal minimum.

The bending induced in the BTOF altered the effective refractive index of the modes involved, and the refractive index profile can be approximated according to the curvature radius, $R_{c}$, by [40,41]:

(2) $$n\left(R_{c}\right)=n_{s}\left(1+\frac{x\left(1+\chi \right)}{R_{c}}\right)$$

where $n_{s}$ represents the effective index of the straight tapered length, the strain is applied along the x axis, χ is the strain-optic coefficient (−0.22 for silica), and $R_{c}$ is the curvature radius. The maximal change of the refractive index estimated from Equation (2) is of order 0.0004. Figure 4a shows the BTOF_1_ interference reflection response when the magnetic field on the magnetic tape is increased. We observed a uniform wavelength shift across the entire spectrum. The redshift of the waveform is attributed to the decrement of fiber bending and the radius of curvature, $R_{c}$. As a result, the phase difference phase increases as the magnetic field increases. 

To make further tests with our sensor, a specific wavelength region was analyzed to show the reproducibility of the fiber signal. In Figure 4b, specific fringe regions were examined to closely analyze the magnetic field effect. Three zones were studied to validate our method. The first zone (Z_1_) was a peak centered on 1533 nm, where we observed a smaller linear shift of the peak position with magnetic field strength. Due to this effect, the Z_1_ region, corresponding to a maximal signal power peak, was limited in terms of analyzing the fiber sensor signal. The second region (Z_2_) represented a clear, linear wavelength shift increment for the applied magnetic field strengths, with a sensitivity extracted from the data around 70 pm/mT. The third region (Z_3_), around the signal minimum, also presented a limited linear increment with the applied magnetic field. It has the same issues as the Z_1_ region; reduced sensitivity in detection was clear for higher magnetic field values. The issues described above are present in several fiber optic sensors that employ a direct analysis of the signal’s phase modulation. 

**Figure 5:**
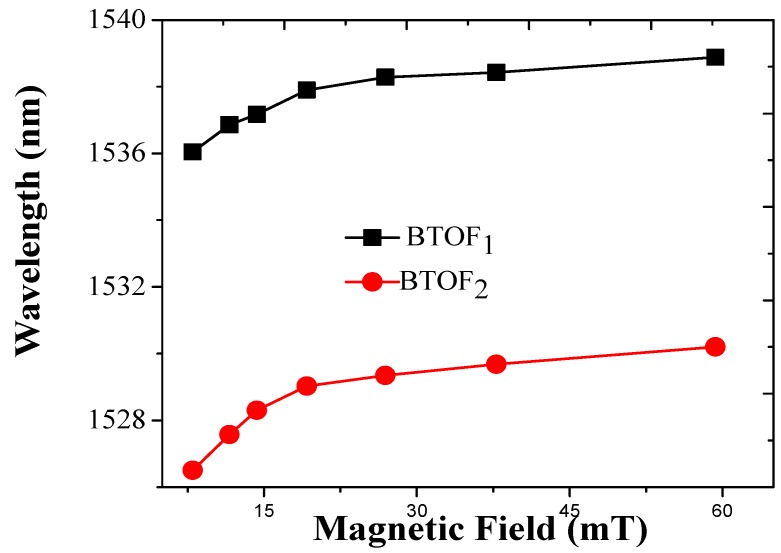
Sensitivities responses for BTOF_1_ and BTOF_2_. Using a signal analysis in zone Z_2_.

The two BTOFs were analyzed by considering the region Z_2_. For BTOF_1_, the initial wavelength was 1536 nm and the maximal sensitivity can be estimated around 70 pm/mT (see Figure 5). Additionally, the BTOF_2_ exhibits a sensitivity around 50 pm/mT. Both sensitivities are an approximation due to the lower linearity exhibited when the magnetic field is applied, this response is related to the magnetic-field–distance association. Indeed, these sensitivities depend on the region and the reproducibility of the fabricated devices. At this point, we detected the magnetic field by inducing curvature over a BTOF; despite the lower linearity, the sensitivity can be compared with other works [7,18,22,24,42,43]. 

### 3.2. Spatial Frequency Signal Analysis

**Figure 6:**
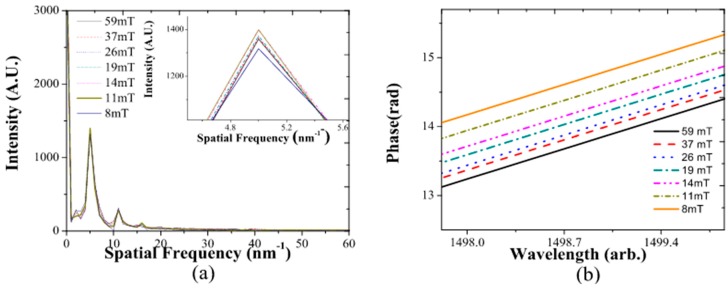
(**a**) Spatial frequency spectrum for BTOF_1_ when the magnetic field was applied; (**b**) phase changes of the filtered BTOF_1_ Fourier component when the magnetic field increased.

In order to detect the magnetic field without the inconveniences mentioned in the wavelength study in the previous section, we employed an alternative signal analysis method using the dominant spatial frequency extracted from the signal spectrum [33]. In our particular case, we used the dominant interference spatial frequency centered at 5 nm^−1^. The two fabricated BTOFs showed the same centered frequencies in Figure 6a. The smaller peak at spatial frequency 10 nm^−1^ was due to variations in the wavelength-dependent signal from a pure sinusoidal wave. For the BTOFs, the dominant (non-zero frequency) Fourier component frequency did not shift when the magnetic field was applied. Besides the constant spectral peak position, small variations in intensity with the applied magnetic field were observed, as shown in the inset in Figure 6a. In addition, these intensity changes were not linear with applied field. To appreciate the magnetic field changes induced in the BTOF_1_ wavelength spectrum, we filtered the dominant Fourier component (see Figure 6a) and its phase was extracted from the data, this phase was then plotted for an arbitrary wavelength without the loss of generality (see Figure 6b) [33]. The phase change in terms of radians was expressed on the y-axis; nevertheless, if the phase shift exceeded 2π, a phase ambiguity sometimes arose. Under gradual changes of the phase, this ambiguity was easily corrected by adding a 2π value when the phase was extracted to unwrap the phase. As observed in Figure 6b, the magnetic field increments could be easily identified by phase changes, and its sensitivity did not depend on the fringe region.

**Figure 7:**
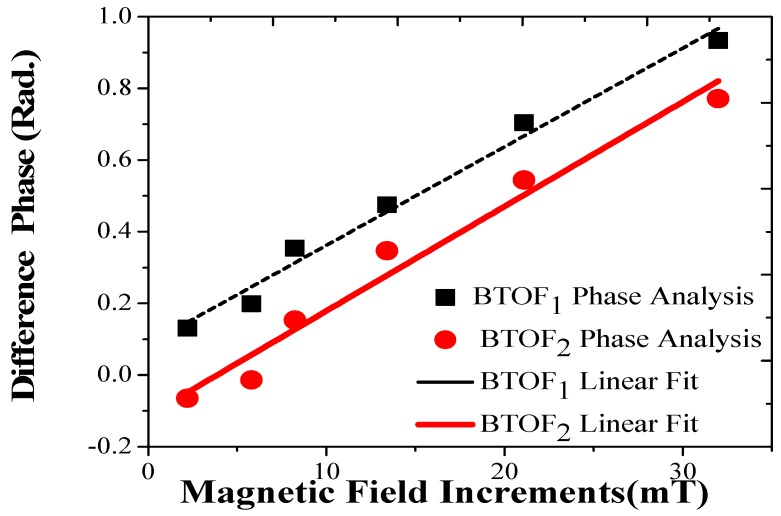
Difference phase analysis of both BTOFs for magnetic field increments.

The difference phase generated was extracted and is shown in Figure 6b; the magnetic field increased and is plotted in Figure 7. Phase change sensitivities of 0.27 rad/mT (BTOF_1_) and of 0.29 rad/mT (BTOF_2_) were calculated. The two BTOF interferometers showed very similar sensitivities, and a clear linear response ($R_{1}^{2}$ = 0.9814 and $R_{2}^{2}$ = 0.9614) was found. Moreover, the phase-matching difference and the power level disparity present in the fabrication process did not affect the sensitivity values. It is important to mention that both sensitivities were obtained by a method that does not require expensive or complicated magnetic materials. In addition, these sensitivities can be changed, as desired, by modifying BTOF physical parameters. However, in this study, we focused on a novel, signal processing method for magnetic field detection, trying to avoid problems presented in the traditional wavelength shifting analysis. 

## 4. Summary

In summary, we constructed a bi-tapered fiber optic sensor to sense magnetic fields using inexpensive transducer material. Our sensor is immune to optical signal polarization changes and requires only a short fiber span to achieve high sensitivity. To demonstrate the sensor design, we characterized two BTOFs with different insertion losses and mismatched phases; moreover, the fiber taper was operated in a double-pass arrangement. Each tapered fiber was analyzed, and they were set and fixed over a thin magnetic tape using translation stages. A magnetic field is then applied using a permanent magnet that was set a distance above the magnetic tape and positioned using a vertical translation stage. As the magnet was placed close to the sensor platform, the magnetic field increased, and the magnetic tape was attracted to the magnet; as a result, the BTOF was bent. The bending of the tapered fiber affected the reflection interference spectrum and a wavelength shifting to longer wavelengths was observed.

Here, three zones were identified and analyzed, and their advantages and disadvantages were explained. The maximal phase shift (wavelength) sensitivity achieved was 70 pm/mT, albeit with lower linearity in the response. The spatial frequency analysis was implemented by filtering the modal interference we found in both tapered fiber spectrums. It is important to stress that, despite the interference reflection differences, the spatial frequency spectrum showed the same dominating modal interference spatial frequency centered at 5 nm^−1^. Once the signal was digitally filtered, its phase is extracted; the magnetic field could be clearly identified from phase analysis. The sensitivity presented in both tapered fibers reached by this method was around 0.28 rad/mT. Moreover, the phase difference as the magnetic field increased showed a linear increment response (R^2^ = 0.9814) to 30 mT. The dynamic range of the sensor could be extended beyond the phase ambiguity point by following the field increments with phase unwrapping, as long as the magnetic material shape change followed the field. Our sensing setup employed low-cost transductor material, without magnetic field detection ambiguity, and had a good linear response. Furthermore, a BTOF for higher or lower magnetic field sensing can be designed using different magnetic materials with stiffer or softer bowing responses to the local magnetic field, giving a range of applications.
